# Interactions between kidney disease and diabetes: dangerous liaisons

**DOI:** 10.1186/s13098-016-0159-z

**Published:** 2016-07-28

**Authors:** Roberto Pecoits-Filho, Hugo Abensur, Carolina C. R. Betônico, Alisson Diego Machado, Erika B. Parente, Márcia Queiroz, João Eduardo Nunes Salles, Silvia Titan, Sergio Vencio

**Affiliations:** 1 School of Medicine, Pontificia Universidade Católica do Paraná, Imaculada Conceição, 1155, Curitiba, PR 80215-901 Brazil; 2 School of Medicine, University of São Paulo, São Paulo, Brazil; 3 Hospital Regional de Presidente Prudente, Universidade do Oeste Paulista, Presidente Prudente, São Paulo, Brazil; 4 Santa Casa de São Paulo, São Paulo, Brazil; 5 Institute of Pharmaceutical Sciences, Goiania, Brazil

**Keywords:** Type 2 diabetes, Diabetic kidney disease, Diabetes complications, Glycemic control

## Abstract

**Background:**

Type 2 diabetes mellitus (DM) globally affects 18–20 % of adults over the age of 65 years. Diabetic kidney disease (DKD) is one of the most frequent and dangerous complications of DM2, affecting about one-third of the patients with DM2. In addition to the pancreas, adipocytes, liver, and intestines, the kidneys also play an important role in glycemic control, particularly due to renal contribution to gluconeogenesis and tubular reabsorption of glucose.

**Methods:**

In this review article, based on a report of discussions from an interdisciplinary group of experts in the areas of endocrinology, diabetology and nephrology, we detail the relationship between diabetes and kidney disease, addressing the care in the diagnosis, the difficulties in achieving glycemic control and possible treatments that can be applied according to the different degrees of impairment.

**Discussion:**

Glucose homeostasis is extremely altered in patients with DKD, who are exposed to a high risk of both hyperglycemia and hypoglycemia. Both high and low glycemic levels are associated with increased morbidity and shortened survival in this group of patients. Factors that are associated with an increased risk of hypoglycemia in DKD patients include decreased renal gluconeogenesis, deranged metabolic pathways (including altered metabolism of medications) and decreased insulin clearance. On the other hand, decrease glucose filtration and excretion, and inflammation-induce insulin resistance are predisposing factors to hyperglycemic episodes.

**Conclusion:**

Appropriate glycaemic monitoring and control tailored for diabetic patients is required to avoid hypoglycaemia and other glycaemic disarrays in patients with DM2 and kidney disease. Understanding the renal physiology and pathophysiology of DKD has become essential to all specialties treating diabetic patients. Disseminating this knowledge and detailing the evidence will be important to initiate breakthrough research and to encourage proper treatment of this group of patients.

## Background

The prevalence and incidence of diabetes mellitus (DM) has increased significantly worldwide, mainly due to a higher prevalence of type 2 DM. Type 2 DM globally affects 18–20 % of adults over the age of 65 years. It is estimated that approximately 285 million people, between 20 and 79 years old, currently have DM, 70 % of whom live in middle- and low-income countries. This increase in type 2 DM (DM2) occurs disproportionately, affecting mainly developing countries, thus bringing enormous challenges in the public health care for these patients. The expectation is for this number to increase by more than 50 % over the next 20 years if preventive programs are not implemented. By 2030, it is estimated that almost 438 million people, or 8 % of the adult population, will have DM [1].

Diabetic kidney disease (DKD) is one of the most frequent and dangerous complications of DM2, affecting about one-third of the patients. In addition to the increasing complexity of outpatient care for patients with DM, DKD results in increased hospitalizations and mortality rates, especially due to cardiovascular complications. DKD also increases the demand for renal replacement therapies, such as dialysis and kidney transplants. The combined economic and social costs of this disease are high and of concern to the world’s health systems.

## Methods

In this review article, based on a report of discussions from an interdisciplinary group of experts in the areas of endocrinology, diabetology and nephrology, we detail the relationship between diabetes and kidney disease, addressing the care in the diagnosis, the difficulties in achieving glycemic control and possible treatments that can be applied according to the different degrees of impairment.

Topics explored include pathophysiology, diagnostic measures, pharmacological and nonpharmacological treatments, and recommendations based on special considerations.

## Discussion

The discussion was divided into topics.

### Pathophysiology of type 2 DM

DM2 is a disease characterized by persistent hyperglycemia, resulting from partial or complete insulin deficiency, and it is associated with a clinical picture of insulin resistance. Recently, other organs have been recognized as being involved in the pathogenesis of hyperglycemia in DM2, and it now known that not only dysfunction of the pancreas, but also of the liver, adipose tissue, intestine, kidneys, and central nervous system may contribute to this hyperglycemic state [2].

Insulin resistance (IR) is one of the pillars dictating the pathogenesis of DM2 and may differ according to body tissues. However, where does IR begin? Some authors argue it starts in the liver, others in the muscle, and others in the brain. What we know is that IR is present in various body tissues (liver, peripheral muscle, central nervous system, adipocytes, etc.) of patients with DM2, preventing glucose to entry into the cell and causing hyperglycemia. Several studies show that insulin has an anorexigenic action in the central nervous system [3–5]. However, the caloric intake in obese individuals is enhanced even in the presence of hyperinsulinemia, suggesting a clinical picture of IR in the brain [2]. Regarding peripheral IR, it is well established that IR directly correlates with deposits of visceral [6] and intramyocellular (within the myocyte) fat [7, 8]. This can be explained by the inflammatory role of adipocytes in producing interleukin-6 and tumor necrosis factor-α, among other pro-inflammatory substances that alter intracellular signaling through the insulin receptor and consequently decrease the expression of glucose transporters of the cell membrane (GLUTs), leading to IR. In the muscle, when deposition of intramyocellular fat occurs, especially in the cytoplasm far from mitochondria, cytoplasmic diacylglycerol production increases, which leads to a decreased membrane expression of GLUT4, subsequent reduction of muscle glucose uptake, and hyperglycemia [7].

Hyperglycemia is not observed in a clinical picture of impaired glucose tolerance or pre-diabetes, since hyperinsulinemia can still compensate for IR and maintain normal levels of blood glucose. When hyperinsulinemia can no longer compensate for IR and insulin secretion begins to decline, the disruption of these variables results in hyperglycemia and a diagnosis of DM. In the early stages of DM2, the clinical picture of hyperinsulinemia persists. However, reduced insulin secretion is mainly responsible for the clinical picture of hyperglycemia. In the later stages of the disease, IR persists. However, the clinical picture, characterized by deficient insulin secretion, worsens, thus exacerbating the loss of glycemic control.

The gold standard to evaluate insulin resistance is the euglycemic insulin clamp technique. However, this technique is difficult to perform, expensive to apply, and is only used in clinical studies [9]. More simply, we can estimate IR using formulas that correlate with the clamp, such as the homeostatic model assessment (HOMA-IR): [fasting insulin (mU/mL) × fasting blood glucose (mmol/L)]/22.5 [10]. It is important to remember that HOMA-IR assesses hepatic IR, as the calculation involves the use of fasting blood glucose and insulin levels. On the other hand, the Matsuda index can estimate hepatic and peripheral insulin sensitivity, using glycemia and insulinemia values obtained through an oral glucose tolerance test [11].

In addition to IR, insulin deficiency is essential to manifest hyperglycemia in DM2. There are several factors involved in the process of insulin secretion, and incretins are one of the most important. Incretins are hormones secreted by the gut that have different functions upon binding to their receptors, expressed in various organs and tissues. After a meal, 60 % of insulin secretion depends on the stimulation of incretin hormones [12]. There are two main incretins: glucose-dependent insulinotropic peptide (GIP) and glucagon-like peptide-1 (GLP-1). Both are involved in glucose homeostasis. However, GLP-1 is more important than GIP, since it also inhibits glucagon secretion, slows gastric emptying, and inhibits hunger, whereas GIP does not [13]. Therefore, GLP-1 is a target of several incretin therapies for the treatment of DM2. GLP-1 is secreted by the L-cells of the ileum and has a half-life of 2 min; it is inactivated by dipeptidyl dipeptidase-4 enzyme [13]. When released into the circulation, GLP-1 binds to its receptor, which is expressed in different tissues, and promotes different actions. The GLP-1 receptor is a G-protein-coupled receptor, and binding activates adenylyl cyclase, leading to a subsequent increase in cyclic adenosine monophosphate, which activates protein kinase A and increases the release of insulin [14]. It is worth noting that incretins stimulate glucose-dependent insulin secretion, i.e., only if blood glucose is elevated. The GLP-1 receptor is expressed in multiple organs besides the pancreas, such as the intestine, kidneys, heart, and central nervous system. It exerts different functions in different tissues: (1) in the central nervous system, it decreases hunger and increases satiety; (2) in the pancreas, after a meal, it stimulates insulin secretion from β cells and decreases the release of glucagon by α cells; (3) in the liver, it reduces glycogenolysis and gluconeogenesis by decreasing postprandial glucagon; (4) in the heart, it plays a cardioprotective role; and (5) in the vessels, it acts as a vasodilator [15].

Patients with DM2 have an impaired incretin system, in addition to the dysfunction of β and α cells. A decreased effect of incretins affects not only the secretion of insulin, but also other beneficial effects promoted via the GLP-1 receptor. This supports several modern proposed DM2 pharmacological therapies aimed at improving the effects of incretins.

In addition to the pancreas, adipocytes, liver, and intestines, the kidneys also play important roles in glycemic control. In the tubular reabsorption of glucose, renal gluconeogenesis also contributes significantly to glucose homeostasis. By 1938, the first studies [16] on the role of the kidney in glycemic control were conducted in animals, and in the late 1950s, studies on renal glucose metabolism were conducted in humans [17–20]. Several hormones are involved in regulating renal reabsorption of glucose: hyperinsulinemia blocks the secretion of renal glucose as it does in the liver [21, 22]. However, epinephrine infusion increases the release of renal glucose [23], and this effect is not altered by glucagon [24]. Although there is not yet available data in humans, several studies suggest that cortisol and growth hormone may stimulate the release of renal glucose [25, 26].

Renal blood flow averages 1000–1500 mL/min, and in healthy individuals, all filtered glucose is reabsorbed by the renal tubules [27]. On average, the kidneys filter 162 g of glucose per day (considering a glomerular filtration rate of 180 L/day); 90 % of this is reabsorbed via the sodium/glucose cotransporter (SGLT) 2 expressed in the proximal tubule. The remaining 10 % of filtered glucose is absorbed by the SGLT1 transporter located in the descending proximal tubule, thus usually preventing glycosuria [2]. In individuals with DM, the Tm for glucose (maximum capacity of renal tubular reabsorption of glucose) is higher than in healthy individuals, thus worsening hyperglycemia. On average, the kidneys contribute 20 % of total body glucose through glucose tubular reabsorption and renal gluconeogenesis [27]. Because the kidneys play a role in glucose homeostasis, therapies have been developed to reduce tubular reabsorption of glucose, which is achieved by inhibition of the SGLT2 transporter. Other therapies suppress both SGLT2 and SGLT1, with the aim of improving glycemia by increasing glycosuria. Unlike previous views regarding glycosuria, after the introduction anti-SGLT1 and -SGLT2 medications in the treatment of DM2, glycosuria has become a desired clinical sign.

### Glucose homeostasis in kidney disease

**Fig. 1:**
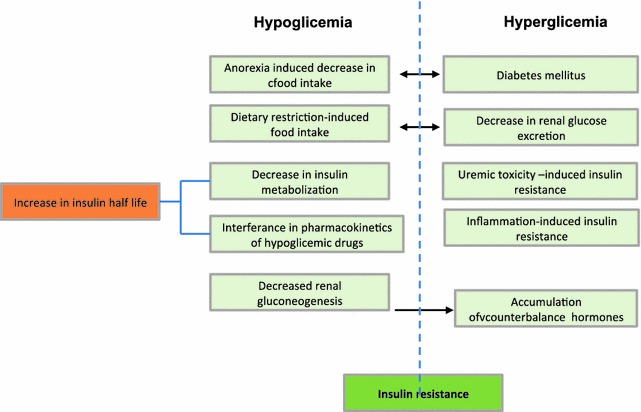
Chronic kidney disease mechanisms predisposisng to hyperglycaemia and hypoglycaemia

Glucose homeostasis is extremely altered in patients with CKD, who are exposed to a high risk of both hyperglycemia and hypoglycemia. Both high and low glycemic levels are associated with increased morbidity and shortened survival in this group of patients. Factors that are associated with an increased risk of hypoglycemia in CKD patients include decreased renal gluconeogenesis, deranged metabolic pathways (including altered metabolism of medications) and decreased insulin clearance. On the other hand, decrease glucose filtration and excretion, and inflammation-induce insulin resistance are predisposing factors to hyperglycemic episodes (Fig. 1). Appropriate glycaemic control tailored for diabetic patients is required to avoid haemodialysis-induced hypoglycaemia and other glycaemic disarrays [28].

### Glycemic monitoring in CKD

As lack of glycemic control increases the rate of progression of renal failure, proper glycemic control in the early stages of CKD is crucial [29]. The United Kingdom Prospective Diabetes Study (UKPDS) provided the first evidence that intensive glycemic control, determined by a more aggressive therapy combined with monitoring and more frequent medical follow-up, could reduce the long-term complications caused by DM2 [30]. Although hyperglycemia is the biochemical marker of DM, hemoglobin A1c (HbA1c) has slowly become the cornerstone for the diagnosis and monitoring of DM since its introduction into routine clinical practice in 1976 [31]. There are confounding factors in the measurement of HbA1c, among which we have previously reported the difference in intracellular-extracellular glucose homeostasis, the survival time of red blood cells (hemolytic anemia), and non-glycemic genetic determinants of hemoglobin glycation. For this reason, the use of HbA1c as the only criterion for the diagnosis of DM in non-Caucasian individuals can lead to misclassification. In addition to its recent role as a diagnostic marker, HbA1c is also used to evaluate the degree of metabolic control in diabetics and to predict the risk of vascular complications [32, 33].

There is conflicting evidence on the role of HbA1c in reflecting long-term glycemic control in patients with CKD. Moreover, the association between glycemic control and outcomes may be different in patients with or without CKD. Uremia causes a unique internal environment, which creates the need to assess each case in a personalized manner. Therefore, markers for monitoring glycemic control, specifically in individuals with CKD, need to be evaluated [34].

Glucose monitoring for the prevention of acute and chronic complications is critical in the management of DM. Therefore, we will discuss the main markers for glycemic control and their limitations in patients with CKD.

#### Blood glucose concentration

According to biological variation, in order to avoid patients misclassification, glucose measurement should have an analytical imprecision ≤2.9 %, a bias ≤2.2 %, and a total error ≤6.9 %. In a perfect scenario, glucose analysis should minimize total analytical error, and methods should be without measurable bias [35].

Enzymatic methods for glucose analysis are well standardized. A survey conducted by the College of American Pathologists (CAP) reveals that hexokinase or glucose oxidase is used in nearly all analyses performed in the U.S. few laboratories use glucose dehydrogenase. Glucose is stable for 8 h in samples collected with an antiglycolytic agent. In plasma, serum, and other liquids already separated from cells, the level of glucose is stable for 3 days at 2–8 °C if there is no bacterial contamination [36].

##### Important factors interfering with glucose measurement

Bilirubin levels >10 mg/dL produce negative interference when the endpoint method is utilized. For samples containing triglyceride concentrations >1100 mg/dL, the turbidity effect can be minimized using by diluting it with NaCl 150 mmol/L (0.85 %) and repeating the measurement. Bilirubin levels ≤10 mg/dL, hemoglobin ≤150 mg/dL, and triglycerides ≤3500 mg/dL do not produce significant interference when the kinetic method is used. Ascorbic acid at concentrations >100 mg/dL also interfere with the reaction, producing falsely low results [36].

##### Preparation for the exam

The collection should be preceded by a fasting period of 8–12 h, with water intake. Physical activity and a habitual diet are recommended the day before the examination as well as a standard diet of 150 g carbohydrates [36].

#### Home glucose monitoring or self-monitoring (SM)

SM is a valuable resource for both the patient and the doctor, as it is undoubtedly among the markers for glycemic control that provide the greatest amount of information about daily nutrition and the resultant glycemic responses. Pimazzoni et al. established several criteria that must be followed in order to produce favorable results, optimizing the use of SM [37]:

- The patient should be instructed regarding the proper use and benefits provided by the correct SM practice.
- The patient should follow a continued practice of DM education, not only in the initial discovery of the disease but also as the disease evolves.
- There is no frequency of tests that can be recommended to all patients. On the contrary, this frequency should be individualized and adapted to the clinical conditions of each patient.
- The SM results should be effectively used by the physician and other health professionals, to promote constant adjustments in the therapeutic conduct and supplementary guidance of nursing, nutrition, psychology, and physical education.

SM does not interfere with monitoring in diabetic patients with CKD. Its limitations include the need for training and economic access to tapes. However, there is no doubt that establishing a pattern of glycemic variation is fundamental. The importance of glycemic variability as an isolated factor for cardiovascular risk is well established [38]. Another important parameter regarding SM is the potential to download the information on specific software, generating accompanying graphs that facilitate understanding and decision-making.

#### Glycated hemoglobin

Glycation is a nonenzymatic reaction of glucose binding to a protein, in this case, to hemoglobin, yielding glycated hemoglobin, or HbA1c. In keeping with this notion, the term glycosylated hemoglobin is incorrect. The generic term “glycated hemoglobin” refers to a group of substances formed from the reactions between hemoglobin A (HbA) and certain sugars. This process is concentration- and time-dependent. In practical terms, this means that the greater the concentration of glucose available, the higher the concentration of HbA1c. In contrast, over time, there is a lower the binding of glucose to hemoglobin [39].

In contrast to plasma glucose, HbA1c represents nonenzymatic glycation, which depends on the concentration of glucose in the intra-erythrocytic compartment. Although several studies found a good positive correlation between the concentrations of HbA1c and glucose in diabetic patients with and without CKD, the variable relationship between HbA1c and estimated average glucose remains a potential source of concern [40].

It is interesting to notice that, normally, 97 % of hemoglobin is HbA. Only 6 % of HbA undergoes a glycation process and becomes HbA1c. Ninety-four percent of HbA undergoes no action induced by any sugars and is called HbA0. In turn, HbA1c is divided into subtypes in accordance with the type of sugar that produces glycation. Twenty percent of HbA1c is influenced by fructose-1,6-diphosphate and glucose-6-phosphate, forming HbA1a and HbA1b. The remaining 80 % of HbA1c is glycated dependent upon glycemic variation and is called HbA1c [39].

##### Main laboratory methods used

The following methods are approved by the National Glycohemoglobin Standardization Program (NGSP): high performance liquid chromatography (HPLC—a method that was applied in the diabetes control and complications trial (DCCT)), boronic acid affinity chromatography, enzymatic, immunoassay, and capillary electrophoresis. Since different methods quantify different ratios of glycated hemoglobin, the results are different. However, an excellent correlation was observed in a sample without hemoglobin variants or the presence of interfering factors. Through NGSP, the values of glycated hemoglobin can be expressed to provide equivalent results of the glycemic status of the patient, regardless of the method used, thus the same criteria can be widely applied. NGSP standardized these methods for the results to be comparable to those obtained by DCCT, in which the relationship between the average levels of blood glucose and the risk for vascular complications was established. A list of methods and worldwide laboratories, the certification of which depends on the demonstration of an acceptable accuracy and compliance with DCCT standards, is also provided on their website (http://www.ngsp.org) [41].

##### Important factors causing technical interference

Interference in the dosage of HbA1c might occur and depends on the method used: factors increasing HbA1c measurements include renal impairment (increased urea binds to hemoglobin, producing carbamylated hemoglobin that interferes with HbA1c measurement); use of acetylsalicylic acid (binds to hemoglobin, producing acetylated hemoglobin, which interferes with HbA1c measurement; usually, this occurs with high doses of acetylsalicylic acid); hypertriglyceridemia; and hyperbilirubinemia. Finally, factors decreasing HbA1c measurements include hemoglobin glycation inhibition factors (e.g., vitamins C and E) [42].

##### Clinical conditions that interfere with the method

Interference might occur with the dosage of HbA1c, depending on the method: factors increasing HbA1c measurements include polycythemia, anemia due to iron deficiency, folic acid, or vitamin B12; chronic alcoholism; and opiates. Factors decreasing HbA1c measurements include conditions that shorten the half-life of red blood cells (e.g., hemolytic anemia, hemorrhages), lead poisoning, erythropoietin deficiency secondary to renal failure, multiple myeloma, hyperthyroidism, leukemia, and severe burns with loss of fluid and proteins [42].

Fasting is not necessary for the collection of the material. Whole blood collected using EDTA as anticoagulant. The blood can be stored in a refrigerator for a week. Heparinized samples should be assayed in a maximum of 48 h [41].

##### Limitations of glycated hemoglobin in CKD

In addition to glucose, other factors might also influence HbA1c: this is the main reason for which the dosage of HbA1c is questioned in patients with CKD. Among these influences, we highlight a few. First, the formation of HbA1c is dependent on the interaction (intensity and duration) between the concentrations of glucose and blood erythrocytes. On average, erythrocytes survive 117 days in men and 106 days in women. At a certain point, a blood sample contains erythrocytes of different ages, mainly younger elements and with different degrees of exposure to hyperglycemia [40]. HbA1c is a measure for the mean level of blood glucose in the past 90 days. The impact of recent blood glucose levels on the measurement of A1c are: 50 % for the last month, 25 % for the 2nd month ago, and 25 % for the 3rd and 4th month ago.

An unexplained discrepancy between HbA1c and other measurements of glycemic control can be partly due to the different life span of erythrocytes. Decreased erythropoiesis, caused by iron or vitamin B12 deficiency or aplastic anemia, leads to an increased number of aged red blood cells and a subsequent progressive increase of HbA1c, unrelated to glycemic control [43].

Anemia due to iron deficiency increases HbA1c up to 2 %, which can be reverted by iron supplementation. Conversely, a decrease in HbA1c is observed after the administration of erythropoietin, iron, and vitamin B12, and in cases of hemolytic anemia. Due to a reduction in the survival of red blood cells, younger erythrocytes have less time to be exposed to a glycemic environment and therefore undergo less glycation [44] Hemoglobinopathies, of which the most common example is sickle cell anemia and thalassemia, can lead to problems in the interpretation of HbA1c. In case of these changes, in addition to the normal HbA0 glycation to form HbA1c, other glycation products derived from HbC (African populations), HbD (indigenous populations), HbE (Asian populations), or HbS (sickle cell anemia) could be formed [43].

In the third National Health and Nutrition Examination Survey, alcohol consumption was associated with low levels of HbA1c in 1024 adults with DM. These findings were confirmed in a large follow-up study of 38,564 adult patients with type 1 or 2 DM. An increase in alcohol consumption predicts lower HbA1c values. Also, the intra-erythrocytes pH can interfere HbA1c. In patients with chronic renal failure, lipid peroxidation of Hb can increase hemoglobin glycation. Chronic ingestion of aspirin and high doses of antioxidants (e.g., vitamins C and E) may reduce HbA1c, since they inhibit glycation. It is unclear whether these phenomena could change clinical practice [45].

In addition to the changes described above, it is important to highlight that new methods detect differently the presence of hemoglobinopathies, and the presence of carbamylated hemoglobin can interfere with the dosage. HbA1c measured by HPLC detects the carbamylated fraction differently than does immunoturbidimetry, which does not identify this fraction; consequently, patients with renal failure present higher levels of HbA1c when measured using HPLC [44].

#### Glycated albumin

The dosage of glycated albumin (GA) is gaining interest as a potential marker of glycemic control. GA is a ketoamine formed by the non-enzymatic oxidation of albumin by glucose. As the half-life of albumin is of approximately 15 days, GA is used as a short-term measurement of glycemic control, that is, 2–3 weeks, and as such, it might act as an intermediate time index of glycemic control [44].

Several methods can be used for the measurement of GA, including affinity chromatography, ion-exchange chromatography, HPLC, immunoassay techniques, capillary electrophoresis, and other electrophoretic and enzymatic assays. It is not influenced by sex, red blood cell life span, or erythropoietin therapy; however, for serum albumin concentration, the results are conflicting [46].

However, the results can be influenced by age, nutritional status, albuminuria, cirrhosis, thyroid dysfunction, and smoking. GA is inversely influenced by body mass index, body fat mass, and visceral adipose tissue [46].

#### Glycated fructosamine

Fructosamine is the generic name given to all glycated proteins, of which albumin is the major plasma fraction, after hemoglobin. Although the dosage of fructosamine can be automated, thus making it cheaper and faster than HbA1c measurement, there is no consensus on its clinical utility [47].

The level of fructosamine correlates better with the average glucose levels over the previous 10–14 days. As this is a measure of total glycated serum proteins, of which glycated albumin represents approximately 90 %, fructosamine concentrations can be influenced by the concentrations of serum proteins and the profile of different proteins [47].

Moreover, fructosamine is influenced by the concentration of bilirubin and substances with low molecular weight, such as urea and uric acid. GF is not modified by changes in the metabolism of hemoglobin. However, it is affected by disturbances in protein turnover. The reference values depend on age, sex, sample population, and test method applied [48].

Unfortunately, the data show conflicting results concerning the correlation between fructosamine and glucose concentrations in patients with CKD. The values may be influenced by nephrotic syndrome, thyroid diseases, administration of glucocorticoids, liver cirrhosis, and jaundice [48].

#### 1,5-Anhydroglucitol (1.5-AG)

1.5-AG is another blood glucose marker and is a naturally occurring dietary plasma polyol, for which levels are maintained constant during normoglycemia by filtration and renal reabsorption. The physiological function and metabolism of 1,5-AG are not well defined. 1,5-AG is a non-metabolizable glucose analog found in plasma after food intake. It is characterized by urinary excretion, filtration through the glomeruli at a rate of 5–10 g/L, and high tubular reabsorption (>99 %), which is inhibited by glucose during periods of hyperglycemia [49].

The levels of 1,5-AG in blood are altered less than 24 h after hyperglycemic episodes, and the repetition of these episodes dramatically decreases its concentration. The values of 1,5-AG reflect hyperglycemia over a period of approximately 1 week. In addition to the glycosuria threshold, the measurement of 1,5-AG could play an adjuvant role in the control of DM, especially as a short-term single marker for hyperglycemia excursions [50].

The relationship between HbA1c and glucose is more complex in more advanced stages of CKD due to the great variability in hemoglobin, nutritional status, and inflammation. Moreover, these underlying comorbidities may also hinder the prognostic value of HbA1c [44].

**Fig. 2:**
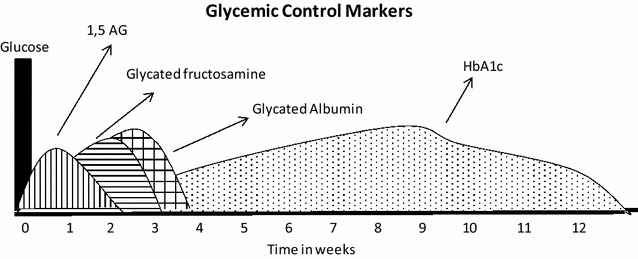
Correlation between each marker and the time of hyperglycemia that each indicates

Figure 2 shows the correlation between each marker and the time of hyperglycemia that each indicates.

Current guidelines recommend using HbA1c as preferred biomarker for glycemic control in patients with CKD, with a goal of 7 % to prevent or delay the progression of microvascular complications of DM, including diabetic nephropathy [33]. However, these guidelines refer mainly to the initial stages of CKD. In diabetic patients with advanced disease, it is suggested that the objective of a very intensive glycemic control, HbA1c <6.5 %, can be associated with increased mortality.

A cohort study assessing 54,757 diabetics on hemodialysis demonstrated that an average HbA1c >8 % or an average glucose >200 mg/dL seemed to be associated with an increased cardiovascular mortality [51]. A recent meta-analysis, investigating the relationship between HbA1c and risk of death in diabetic hemodialysis patients, showed that the level of HbA1c remains a useful clinical tool for the prediction of the mortality risk [52].

Although glycated albumin presents advantages in patients with CKD, several authors argue that CKD is characterized by the disruption of albumin homeostasis and that the threshold of serum albumin for which the risk of death increases varies according to the dialysis modality [53]. In the presence of hypoalbuminemia, plasma protein glycation is increased. However, glycated albumin seems to reflect the percentage of albumin that is glycated, regardless of the concentration of total serum albumin, although more studies on a large scale with dialysis patients would be required to confirm this observation [54].

Glycated albumin seems to be a better marker to reflect the accuracy of glycemic control when compared to HbA1c in patients with DKD. However, due to limited data, the absence of studies on the results of interventions based on glycated albumin and its expensive and laborious methodology, indicate that it might be premature to abandon HbA1c in favor of glycated albumin [55].

Thus, our recommendation is that diabetic patients with CKD would be monitored in the best possible way, in the attempt to prevent the progression of the disease and an increase in complications. Therefore, we suggest monitoring HbA1c every 3 months, which can be associated with home SM when possible. Other exams such as glycated fructosamine, glycated albumin, and 1,5-AG could be used as additional tools, rather than replacing HbA1c.

### General approach of DM treatment in CKD

#### General considerations for the control of DM in CKD

Glycemic control is fundamental in the prevention and progression of complications associated with DM [56, 57]. Studies show that reducing HbA1c to values ≤7 % influences the reduction of microvascular complications caused by DM, and if implemented early, it is also associated with a reduced occurrence of macrovascular complications [56, 57].

The goals proposed by the Brazilian Diabetes Society (SBD) in 2013/2014 Guidelines recommend achievement of the following aims: fasting glucose <100 mg/dL, preprandial glycemia <130 mg/dL, postprandial glycemia ≤160 mg/dL [58], and HbA1c <7 %. In 2015, The American Diabetes Association (ADA) reinforced its proposal to keep HbA1c optimal values <7 % for most diabetic adults [59]. However, in recent years, the associations focused on the treatment of DM have systematically reviewed the optimal values of glycemia and HbA1c goals for diabetic patients, with the aim to define individualized objectives to prevent the onset of chronic complications, aiming also to reduce the occurrence of hypoglycemia.

The ACCORD (action to control cardiovascular risk in diabetes) trial was a landmark in demonstrating that patients with high cardiovascular risk, when treated intensively with the aim to achieve HbA1c of approximately 6 %, presented an increased risk of death [60]. After this study, associations such as ADA began to recommend individualized HbA1c goals for patients with a history of severe hypoglycemia, limited life expectancy, patients with microvascular or macrovascular complications in advanced stages, and patients with multiple comorbidities. The recommendation of less strict HbA1c goals (around 8 %) for these groups aims to reduce the morbidity and mortality associated with a very strict glycemic control, often related to an increase in hypoglycemic episodes [59].

Specifically in relation to DKD, classical studies have also previously demonstrated that improved glycemic control is associated with a reduced incidence of albuminuria in both type 1 and type 2 DM [56, 57]. Even in secondary prevention, i.e., when the kidney disease is already established, glycemic control remains a major therapeutic weapon to combat the progression of CKD [61, 62]. The ADVANCE (action in diabetes and vascular disease) trial showed that intensive control was able to reduce albuminuria, nephropathy, and the need for hemodialysis [63]. Similarly, the ACCORD trial showed a significant reduction in albuminuria (although not in advanced renal disease) in the group treated with an intensive therapy for glycemic control [60].

However, despite evidence correlating the optimization of glycemic control to the benefits observed in the evolution of DKD, glycemic and HbA1c objectives are very difficult to define and achieve in this population. The complexity of glycemic control in this group of patients is explained not only by the metabolic alterations associated with DKD, but also the specificity and greater difficulty in the use of hypoglycemic drugs, difficulty in monitoring glycemic levels, behavioral addictions related to years of DM and a fear of hypoglycemia, as well as sociocultural and economic factors.

DKD progresses with several metabolic changes, which occur concomitantly with the progressive decline in glomerular filtration rate (GFR). Using the euglycemic insulin clamp, DeFronzo et al. showed that the glucose used by peripheral tissues in response to insulin is reduced in uremia [64]. The increased insulin resistance is related to the accumulation of uremic toxins, markers of chronic inflammation, increased visceral fat, oxidative stress, and vitamin D deficiency. Progression to uremia is associated with decreased insulin sensitivity of peripheral tissues, increased hepatic gluconeogenesis, decreased glucose uptake by skeletal muscle cells, and deficiency of intracellular glycogen synthesis and subsequent hyperglycemia [65]. On the other hand, the risk of hypoglycemia is a constant concern, since this is increased in diabetic patients with CKD. The pathogenesis of hypoglycemia in these patients is related to changes in glucose metabolism, decreased insulin degradation, and changes in the metabolism of hypoglycemic agents. With a progressive reduction in GFR, we observed a decrease in the clearance of oral hypoglycemic agents, and sometimes, a longer time of action of these drugs and their active metabolites. Similarly, insulin metabolism is also altered, since part of its metabolization and excretion is carried out by the renal system [66–68]. A restricted diet, either by prescription or even due to uremia, reduces hepatic gluconeogenesis, thus contributing to the occurrence of hypoglycemic episodes observed at higher frequency in this population [69, 70].

Therefore, since CKD is a condition that increases predisposition to hyperglycemic and hypoglycemic peaks, the choice of drug treatment for these patients should be carefully considered [71–73]. Most classes of oral hypoglycemic agents should be avoided when GFR is <40 mL/min, which indicated a higher risk of hypoglycemia. Insulin is the therapy of choice for the treatment of diabetic patients with advanced CKD, and for insulinization to occur properly. Adherence and understanding of patients are of utmost importance. In phases IV and V of CKD, almost all patients with DKD (in which DM is the central determinant in the etiology of DKD) need insulin. Patients with advanced CKD in which DM is another comorbidity, rather than the etiology of CKD, require insulin less frequently. Therefore, it is important that attending physicians have a broad knowledge of the arsenal of oral hypoglycemic agents that are currently available, in order to avoid the use of insulin when possible and the inappropriate and dangerous use of oral hypoglycemic agents. Oral hypoglycemic agents could also be used in patients with burnout syndrome, in which the “disappearance” of DM is almost always observed because of important homeostatic changes related to diet restrictions, catabolism, weight loss and greater circulation of endogenous insulin.

In any case, most patients with advanced CKD need to use insulin for the safe and effective control of DM. However, for this to be achieved, a number of points should be discussed with the patient and the family:

- Proper storage of insulin
- Application techniques, insulin mixing techniques, and rotation of daily application locations
- Strict diet at pre-determined times
- Guidelines on how to proceed in the presence of hypoglycemia
- Adherence to multiple daily insulin injections
- Conduction of pre- and postprandial capillary blood glucose tests, also conducted at dawn, facilitating dose adjustment.

These guidelines require a commitment not only from the patients and their families, but also from a multidisciplinary team to make certain that the procedures are fulfilled. It is known that many diabetic patients who evolve towards a progressive loss of renal function have a personal history of poor adherence to the treatment, either due to inherent factors of the patient or the difficulty of the health system in dealing with a complex framework, thus demanding specific care. We also noticed that many patients with advanced stage kidney disease often have comorbidities that further hamper their adherence to the treatment. Patients with diabetic retinopathy (DR) or those who have undergone amputation require the support of their families for periodic consultations, drug administration, and completion of capillary blood glucose monitoring tests.

The awareness and motivation of the patients and their families to complete the proposed treatment strategies in order to achieve the necessary goals for proper metabolic control should always be reviewed and emphasized by the multidisciplinary team. It is important that the entire team pays attention in identifying the problems that can range from understanding the subject, to access to information and inadequate use of insulin. These habits are particularly common in patients with a history of poor glycemic control caused by self-medication for many years or by extreme fear of hypoglycemic episodes that led to the use of lower doses of insulin (most often not disclosed to the medical team). A condition often observed in populations of lower socioeconomic conditions is concurrent very high glycated hemoglobin levels and frequent episodes of hypoglycemia. Therefore, the best option is to provide DM re-education, review dietary patterns, and ensure fractionation of insulin dosage. Often, however, the medical team responds inadequately, and insists on increasing the insulin dose, which the patient reduces without reporting the decrease because of fear of worsening hypoglycemia. This creates a complete dissociation between the healthcare team and patient, with mutual loss of trust and overall inefficacy of the treatment. If this occurs, the process of re-education becomes even more important, since in addition to directly approaching patients and their families, it becomes necessary to work on concepts, insecurities, and prescription patterns of the attending medical team.

According to NKF–KDOQI (National Kidney Foundation–Kidney Disease Outcomes Quality Initiative), HbA1c objectives in diabetic patients with CKD do not differ from those recommended for patients without renal disease, aimed to maintain HbA1c values lower than 7 % [74–76]. However, as already mentioned, the importance of individualization of HbA1c goals has already recognized by the ADA [59]. It is noteworthy that most diabetic patients with CKD or DKD, in a broad sense, fit the ADA’s criteria for high risk of hypoglycemia.

#### Hypertension, dyslipidemia, and other microvascular complications in diabetic patients with CKD

Blood pressure control is fundamental in the management of kidney disease progression. In general, diabetic patients with lower blood pressure levels and renal disease tend to experience slower progression of the pathology compared to hypertensive patients with the same condition [77]. Non-pharmacological measures (dietary changes and increased physical activity) have an impact on blood pressure control and should be encouraged. Drugs inhibiting the renin-angiotensin system through its specific renoprotective effect, regardless of the reduction in systemic blood pressure, have a well-established role in diminishing albuminuria and DKD progression [78].

Studies comparing the effect of angiotensin-converting enzyme (ACE) inhibitors and angiotensin II receptor blockers (ARBs) reported similar effectiveness. Therefore, ACE inhibitors or ARBs are recommended in patients with CKD, regardless of their ethnicity, as first-line treatment or in combination with another antihypertensive drugs [79]. Dose adjustment for these agents should be gradual, with periodic assessment of renal function and potassium levels, since there is a risk for creatinine level elevation and hyperkalemia. Greater attention must be paid to monitoring elderly patients and individuals with advanced stage CKD. In December 2013, the 8th Joint National Committee of Hypertension discussed new strategies for blood pressure control, and it was recommendation that ACE inhibitors and ARBs should not be used in the same patient simultaneously due to the following concerning findings: first, the VA-NEPHRON D trial [80] was prematurely terminated because of concerns about a high prevalence of hypotension, hyperkalemia and acute kidney injury with dual renin-angiotensin system (RAS) therapy. Actually, these adverse events could have been prevented by avoiding forced ACEi up-titration in patients with an eGFR as low as 30 mL/min/1.73 m2 on top of full-dose ARB. Notably, at study closure dual versus single RAS inhibition had already reduced end-stage renal disease events by 34 %, a treatment effect never reported before in type 2 diabetes. Risk reduction was associated with a significantly greater decline in proteinuria and approached nominal significance (P = 0.07) over just 2.2 years of follow-up. Second, in the RENAAL study [78], performed in type 2 diabetic patients, the larger antiproteinuric effect of losartan was associated with a similar (28 %) end-stage renal disease reduction compared to placebo. The treatment effect was, however, not still appreciable at 2.2 years, but became statistically significant over the planned 3.2 years of follow up. These data strongly suggest that also in the VA NEPHRON-D trial, end-stage renal disease events could have been significantly reduced over the initially scheduled 5-year study period. Consistently, the results of a recent meta-analysis showing that dual RAS blockade with ACE inhibition and ARB is the most effective strategy to prevent end-stage renal disease in patients with diabetes and kidney disease [81].

The development of objectives to achieve adequate blood pressure levels to reduce cardiovascular events and progression of kidney disease has been the goal of recent studies. The ACCORD trial failed to show a reduction in cardiovascular events; moreover, in the ACCORD study [77], there were significantly more instances of an eGFR less than 30 mL/min/1.73 m^2^ in the intensive-therapy group than in the standard-therapy group (P < 0.001), although only 38 participants in the intensive-therapy group and 32 in the standard-therapy group had two or more instances of eGFR <30 mL/min/1.73 m^2^ (P = 0.46). The frequency of macroalbuminuria at the final visit was significantly lower in the intensive-therapy group than in the standard-therapy group, and there was no between-group difference in the frequency of end-stage renal disease or the need for dialysis. In addition, the INVEST study also showed no mortality reduction in patients with a desired systolic blood pressure <130 mmHg compared to that in patients with systolic blood pressure 130–139 mmHg [82].

Optimal blood pressure values have not been established. However, in 2015, the ADA aligned its recommendations with hypertension guidelines, recommending the maintenance of a systolic blood pressure lower than 140 mmHg and diastolic pressure below 90 mmHg as goals for the treatment of hypertensive diabetic patients [59]. Similarly, the 8th Joint National Committee of Hypertension also recommends that blood pressure for diabetic patients and individuals with CKD should be <140/90 mmHg [79].

Additional positive phase 2 clinical studies with drugs that have hemodynamic actions such as endothelin antagonists and mineralocorticoid receptor antagonists have led to larger phase 3 trials with atrasentan and finerenone, respectively, in order to address if these drugs indeed delay the development of end-stage renal disease [83]. Positive findings with respect to new glucose-lowering agents such as sodium-dependent glucose transporter 2 inhibitors may lead to a change in the way we treat diabetic individuals with or at risk of DKD. A number of other pathways are currently under active preclinical investigation and hopefully over the next decade will lead to promising drug candidates for subsequent clinical trials [83].

DM and CKD present a significant correlation with increased cardiovascular risk. The risk of events in patients with CKD is considered equivalent to that in patients with a history of coronary disease. Therefore, the combination of these two conditions classifies the patient with DKD as presenting a very high risk for a cardiovascular event. Considering the exacerbated cardiovascular risk of these patients, kidney disease: improving global outcomes (KDIGO) does not recommend the use of routine low-density lipoprotein (LDL) cholesterol level testing to identify patients to be treated or the objectives of the treatment [84].

The current recommendation indicates the use of statins as drugs of choice since their efficacy in primary and secondary prevention of cardiovascular events has been proven, regardless of LDL levels [76, 84]. However, the appropriate dosage remains controversial. While ADA recommends the use of statins in high doses for diabetic patients with risk factors for cardiovascular disease, KDIGO recommends the reduction of the dosage of statins in individuals with a GFR lower than 60/mL/min/1.73 m^2^ [59, 76, 85, 86]. This recommendation is based on the reduction of renal excretions (valid for some statins) and associated comorbidities. However, no studies have shown an increase in adverse events using high doses of statins, and the prescription information of atorvastatin states that there is no need for a dose adjustment in patients with CKD [85]. On the other hand, it is known that patients with CKD have an increased risk of muscle damage with the use of statins, therefore this group of patients should be monitored more carefully. Results of studies on the use of statins in individuals undergoing dialysis, in whom the cardiovascular risk is very high, have been disappointing. Despite the high risk, the cardioprotective effect of statins seems to be less efficient than in other populations. Therefore, the systematic use of statins in dialysis patients is not currently recommended, due to the lack of observed benefits of this intervention in different studies. However, diabetic patients on dialysis continue to receive this drug due to the extrapolation of the proven benefits of statins in the diabetic population in general.

DR (diabetic retinopathy) is a microvascular complication that can occur in type 1 and type 2 diabetic patients, and its prevalence is closely related to the duration of the disease. The prevalence of this complication increases with the duration of DM, affecting more than 60 % of patients with DM2 and more than 90 % of patients with type 1 DM after 20 years of illness [87]. DR is the most frequent cause of blindness in adults aged 20–74 years. The pathogenesis of DR is directly linked to chronic hyperglycemia, and diabetic kidney disease is an important factor for an increased risk of DR incidence. DR and diabetic nephropathy are the two most common microvascular complications in patients with DM; however, whether these complications are only related or directly affect each other, or if their progression necessarily occurs simultaneously, is unclear [88].

Diabetic patients can eventually develop proteinuria, without the presence of DR, or might proliferative DR without the presence of albuminuria. Klein et al. studied a group of normoalbuminuric patients with type 1 DM and found that 36 % of these individuals did not develop DR, 53 % had nonproliferative DR, 9 % had moderate to severe DR, and 2 % had severe DR [89]. On the other hand, the prevalence of DR in patients with diabetic nephropathy and macroalbuminuria is between 70 and 90 %. Proliferative retinopathy is already considered a predictive factor for macroalbuminuria in type 1 diabetic patients. Some authors consider both microalbuminuria and DR to be predictor factors for the progressive loss of kidney function [90].

ADA recommends periodic fundus examinations for retinopathy to be treated in a timely manner, before it progresses to irreversible vision loss. Examinations should be conducted at least annually and can be conducted more frequently depending on the degree of retinopathy [58].

#### Diabetic cardiovascular autonomic neuropathy

Diabetic autonomic neuropathy is a severe complication of DM and is associated with increased morbidity and mortality and decreased quality of life of the patients. Diabetic autonomic neuropathy can affect different systems. Diabetic cardiovascular autonomic neuropathy (DCAN) can manifest clinically as resting tachycardia, severe orthostatic hypotension, syncope, ischemia and asymptomatic myocardial infarction, systolic and diastolic left ventricular dysfunction, increased risk for CKD, stroke, hyporesponsiveness to hypoglycemia, and sudden cardiac death [91].

The association between DCAN and kidney disease is also well established and corroborates with the increase in mortality rates in diabetic patients with CKD. Ewing et al. found an upto 53 % increased mortality in diabetic patients with autonomic neuropathy, compared to 15 % in diabetic patients with no dysautonomia. Moreover, half of all deaths in patients with autonomic neuropathy in this study occurred due to impaired renal function, with 29 % of these being sudden death [92, 93]. In the literature, the prevalence of autonomic neuropathy varies between 21 and 73 % in the diabetic population. The prevalence of autonomic neuropathy ranges from 20 to 80 % in patients with DKD, and affects 66 % of patients with advanced kidney disease and 50 % of patients on dialysis [94]. A recent study showed that DCAN presents an important relationship with CKD, albuminuria, and decline in renal function in patients with DM2 [95].

Treatment of dysautonomic manifestations is essentially symptomatic. Special attention should be given to the intensification of glycemic control, with monitoring of hypoglycemia and changes in lifestyle, including diet and exercise [96]. Regarding drug treatment, fludrocortisone and the *α*1-adrenergic agonist midodrine are considered the drugs of choice in the treatment of DCAN. Erythropoietin is also considered a possible adjunctive drug to increase blood pressure through an increase in the number of erythrocytes and central blood volume, correction of anemia in patients with severe dysautonomia, and neurohumoral effects on wall and vascular tone.

#### Diabetic genitourinary autonomic neuropathy

Almost half of the patients with DM develop some degree of bladder dysfunction. This prevalence may be even higher in populations with advanced CKD who have DM for a long time, or it may be due to the uremic syndrome per se. Bladder dysfunction might result in varying degrees of impairment, ranging from a mild decrease in bladder sensitivity, reduced emptying perception, and alteration in contractility, to situations where there is an increase in bladder capacity, urinary retention, increased frequency of urinary tract infections, lithiasis, and renal failure [97].

The prevalence of sexual dysfunction in patients with CKD can range from 9 % in pre-dialysis patients to 70 % in dialysis patients [98]. In diabetic patients, erectile dysfunction occurs in 35–75 % of patients, 10–15 years earlier than in non-diabetics. In diabetic patients with CKD, the most common causes of erectile dysfunction are organic and are due to vascular disease and neuropathy.

The initial treatment approach for erectile dysfunction in diabetic patients should be glycemic and metabolic control of other associated complications. Specific measures of treatment include drug therapy (group of phosphodiesterase inhibitors: sildenafil, vardenafil, and tadalafil). Intracavernous or intraurethral drugs (papaverine, phentolamine, and prostaglandins) are also used, as well as penile prostheses and vacuum devices [96, 99]. However, the use of these drugs requires a more careful evaluation of CKD because of an increased risk of arrhythmias and heart failure.

### Nutritional recommendations for diabetic patients with CKD

As diabetic patients experience progressive loss of renal function, nutritional issues become more complex. On one hand, in addition to the existing limitations associated with DM, specific restrictions are needed for patients with CKD, including restriction of protein, phosphorus, and potassium. On the other hand, patients with worsening uremic syndrome have a higher risk of protein-calorie malnutrition that needs to be identified and addressed by the medical team. Thus, nutritional monitoring is of utmost importance in this patient population. Standardized protocols should be avoided, and individualized care and monitoring of patients should be implemented.

Initially, patients should be evaluated based on their standard intake and clinical laboratory results. Then, a nutritional counseling plan should be designed based on nutritional guidelines that aid in the development of appropriate diets for patients, always considering individual needs.

For the population of diabetic patients with CKD in the non-dialytic phase, the composition of macronutrients in the nutritional plan is described in Table 1

**Table 1:** Dietary plan macronutrient composition for DKD in the non-dialysis stage. Source: adapted from the Brazilian Diabetes Society (2014)

Macronutrients	Recommended intake/day
Total carbohydrates	45–60 % of TEI (total energy intake)
Saccharose	Up to 10 %
Fructose	Not recommended its addition to food
Dietary fibers	Minimum of 20 g/day or 14 g/1000 kcal
Total fat	Up to 30 % of TEI
Saturated fatty acids (SFA)	<7 % of TEI
Trans fatty acids (TFA)	≤2 g
Polyunsaturated fatty acids (PUFAs)	Up to 10 % of TEI
Monounsaturated fatty acids (MUFA)	Supplemented individually
Cholesterol	<200 mg/day
Proteins	0.8–1.0 g/kg/day in the early stages of disease and <0.8 g/kg/day in the final phases

For patients with DKD, the ADA (2013) recommends a normoproteic diet (0.8–1.0 g/kg/day) in the early stages of CKD and <0.8 g/kg/day or <0.6 g/kg/day in the later stages of the disease (Table 1), with 50 % of protein intake presenting high biological value. Protein restriction aims to act simultaneously as a renoprotective measure, reducing both proteinuria and generation of protein catabolic waste.

For patients with proteinuria >3 g/day, a low protein diet (0.6 g/kg/day) is recommended, as well as the replacement of 1 g of high biological value-protein for each gram excreted. It is important to highlight that provision of a diet low in protein should ensure an adequate energy supply. The recommendation for calorie intake is the same for patients with CKD without the presence of DM—30–35 kcal/kg/day. For overweight and obese patients, the calorie intake recommended should be individualized, although it should not be <25 kcal/kg/day. Regarding glycemic control, the recommended amount of carbohydrates follows the recommendations for the general population (Table 1). The quantity and quality of carbohydrates in the diet and its effects on glycemic responses are well established. Sucrose, when consumed in amounts equivalent to that of other carbohydrates, increases blood glucose level equivalently; therefore, sucrose can be consumed in a nutritionally healthy diet as long as its intake does not exceed 10 % of daily caloric consumption. The use of sweeteners, although indicated, is not essential for the treatment of DM. The use of sweeteners can provide beneficial effects, such as weight loss in overweight or obese patients, due to their low caloric value, thus also reducing insulin.

In addition, adequate intake of food rich in complex carbohydrates (dietary fibers) should be encouraged, since this consumption is associated with glycemic control, satiety, and lipid absorption, thus also contributing to weight control. Although consumption of dietary fibers, especially in the soluble fraction, should be encouraged, it is important to highlight that, in general, foods rich in this nutrient fruits, vegetables, and legumes—are also sources of potassium, a mineral for which intake should be controlled in patients with CKD. Main sources of dietary fibers with low potassium levels are fruits such as pineapple, apple, pear and strawberry, and vegetables such as carrot, watercress, lettuce, escarole, cucumber and cabbage.

One method that can be used to control blood glucose in these patients is carbohydrate counting, in which grams of this macronutrient obtained from meals are recorded throughout the day. This method is efficient in food control and the use of insulin, and its orientation should be individualized.

The recommendations for lipid consumption in diabetic patients are the same as those for individuals with cardiovascular diseases (Table 1), since both patients are at high risk for cardiovascular events. According to the Guidelines of the SBD (2014), the goals for lipid control in diabetic nephropathy include serum LDL cholesterol levels <100 or <70 mg/dL in the presence of any cardiovascular disease and levels of serum triglycerides <150 mg/dL and high-density lipoprotein cholesterol >40 mg/dL for men and >50 mg/dL for women.

In an interesting study conducted by Cardenas et al. (2004) [100], it was identified that, in patients with DM with different degrees of renal disease, a greater intake of polyunsaturated fatty acids and lower intake of saturated fatty acids, as well as a higher ratio of unsaturated and saturated fatty acids, promoted a better evolution of diabetic nephropathy. In the same study, it was found that patients with worsening symptoms consumed higher amounts of saturated fatty acids during the 7-year follow-up.

Considering that arterial hypertension is a factor for the progression of diabetic nephropathy, blood pressure control is essential for the treatment of the disease. The American Dietetic Association (2013) recommends a sodium intake <1500 mg/day, which corresponds to 3.75 g/day of salt. In a study conducted by Houlihan et al. (2012) [101], a diet with 1.2–1.7 g of sodium promoted similar effects to the inclusion of a second antihypertensive drug in the treatment of hypertension.

### Pharmacological treatment: non-insulin antidiabetic agents

Control of blood glucose levels in diabetic patients with CKD in different stages is not adequately standardized. Due to the increased risk of hypoglycemia [102] in these patients, insulin has been considered the safest antidiabetic agent. However, new non-insulin antidiabetic agents proved to be safe and effective. New revisions and guidelines are being published to guide the glycemic control of patients with CKD [70, 103, 104]. Regarding the therapeutic goals, the benefits of strict control of blood glucose levels in recently diagnosed diabetic patients [105] is not observed in diabetic patients with the disease for a long time and who have already developed cardiovascular complications [60, 63, 106], typical of diabetic patients with CKD. For example, the ACCORD trial noted an increase in the overall mortality of 22 % in diabetic patients with the disease for an average of 10 years with a history of cardiovascular diseases, who received intensive glucose control, and aimed for HbA1c of 6.5 % compared to that in the control group, which was to seeking to achieve HbA1c of 7.3 %. This is explained by the increased risk of hypoglycemia episodes associated with a more intensive management of DM and the fact that these patients are more susceptible to the deleterious effects of hypoglycemia, such as activation of the sympathetic nervous system. However, there are benefits of controlling blood glucose levels in diabetic patients with CKD in terms of reducing mortality rates [107], inhibiting progression of CKD [107, 108], and diminishing albuminuria [108]. Nevertheless, the therapeutic goals should be individualized and it should be considered that HbA1c overestimates glycemic control in patients with CKD [109]. Below we discuss several aspects related to CDK of noninsulin antidiabetic agents that are not available in our environment.

#### Metformin

Metformin acts primarily in the liver, decreasing the production of hepatic glucose. Therefore, it is associated with low risk of hypoglycemia. This drug has been used for several years and has proven to reduce cardiovascular events [105] and contribute to mild weight reduction. Thus, it is considered the first choice drug in the treatment of DM2 [110]. This drug is excreted by the kidney and therefore, in patients with CKD, it may accumulate and increase the risk of lactic acidosis, which is a side effect of this drug. Hence, several authors do not recommend the use of metformin in women with creatinine levels >1.4 mg/dL and men with levels >1.5 mg/dL [111]. Others recommend halving the dose in patients with a creatinine clearance of 30–45 mL/min/1.73 m^2^ and suspension of the drug in patients with <30 mL/min/1.73 m^2^ [112]. The relationship between metformin accumulation and lactic acidosis is not well documented [113]. Factors such as acidosis, hypoxia, infection, and dehydration are also associated with the advent of lactic acidosis in patients receiving metformin, and in these situations, the drug should be suspended temporarily.

#### Sulfonylureas

Sulfonylureas act in pancreatic β-cells, releasing insulin. The effectiveness of the class depends on the stores of β-cells, which decreases with the length of the DM. The action of these drugs is independent of glucose levels. Therefore, hypoglycemic episodes are more severe and frequent with the use of sulfonylureas [114]. In patients with CKD, the use of short-acting sulfonylureas metabolized in the liver, including glipizide, gliclazide, and glimepiride, is recommended. However, the use of this class should be avoided in patients with creatinine clearance <45 mL/min/1.73 m^2^. Sulfonylureas can bind to proteins and are not eliminated by dialysis.

#### Glinides

Similarly, glinides, such as repaglinide and nateglinide, act in pancreatic β-cells, releasing insulin. However, these drugs have a shorter half-life and cause less hypoglycemia [115]. Glinides are metabolized predominantly in the liver. These drugs should be used three times a day before meals and can be used in patients with a creatinine clearance <30 mL/min/1.73 m^2^, although with care and a reduced dosage [103].

#### Glitazones

Glitazones, such as pioglitazone and rosiglitazone, increase insulin sensitivity in muscle and adipose tissues by acting on PPAR-ɣ receptors. These drugs are metabolized in the liver, do not accumulate in CKD, and do not cause hypoglycemia, even in patients undergoing dialysis. They are associated with water and salt retention, which limits the use of this class in CKD. It has been shown that the use of rosiglitazone is associated with an increased risk of myocardial infarction [116] and increased cardiovascular mortality in patients undergoing hemodialysis [117]. Therefore, pioglitazone has been used more frequently. Glitazones are also associated with a higher risk of fractures and bladder cancer. Despite the low risk of hypoglycemia, this class of drugs should be avoided in patients with CKD.

#### Alpha-glucosidase inhibitors

Acarbose acts in the gut by inhibiting alpha-glucosidase, the enzyme responsible for digesting carbohydrates. It does not cause hypoglycemia. Its main side effect is flatulence. In CKD, its use should be avoided, since it accumulates and can cause hepatotoxicity [118].

#### Sodium-glucose cotransporter type 2 inhibitors

In the glomeruli, about 180 g of glucose per day is filtered, and nearly all is reabsorbed in the S1 segment of the proximal tubule by sodium-glucose cotransporters. Of these, type 2 cotransporters are the most important [119]. Drugs that inhibit this transporter have been developed, such as dapagliflozin, canagliflozin, and empagliflozin. These drugs block reabsorption of glucose and sodium in the proximal tubule, contributing to improved glycemic control, with no risk of hypoglycemia, as well as hypertension control, due to increased natriuresis. The use of these drugs is associated with a higher incidence of genital infection. This hypoglycemic class is not indicated in patients with a creatinine clearance <45 mL/min/1.73 m^2^ [120], but recent studies show a potential application in the lower (30 ml/min) GFR range. Recent data suggests cardiovascular benefits of this class, opening opportunities for a broader application of SGLT-inhibitors [120].

#### Peptide-1 receptor agonists similar to glucagon (GLP-1 RA)

GLP-1 is an incretin secreted in the gastrointestinal tract in response to food intake. It acts on pancreatic β-cells, releasing insulin, and in pancreatic α-cells, inhibiting the secretion of glucagon in a glucose-dependent manner; therefore, GLP-1 controls blood glucose with a lower risk of hypoglycemia. Moreover, it slows gastric emptying and decreases appetite through a central mechanism, thus contributing to weight loss. GLP-1 receptor agonists, such as exenatide and liraglutide, are peptides with a structure similar to endogenous GLP-1. However, these drugs are resistant to enzyme dipeptidyl peptidase-4 catabolism. The route of administration is subcutaneous. Since they are peptides, they are filtered in the glomeruli and degraded in the proximal tubules, similar to the process associated with insulin. There is little knowledge regarding this class of antidiabetic drugs in CKD, although gastrointestinal effects are exacerbated in patients with CKD, including nausea, vomiting, and diarrhea. Moreover, there have been reported cases of acute renal injury with the use of exenatide in patients with CKD [121]. Therefore, while acquiring further knowledge about this class, it is suggested that careful attention be paid to their use in patients with creatinine clearance 45–60 mL/min/1.73 m^2^. Also, its use should be avoided in patients with a creatinine clearance <45 mL/min/1.73 m^2^ [103].

#### Dipeptidyl peptidase-4 (DPP-4) inhibitors

DPP-4 is an enzyme that degrades GLP-1 and GIP incretins. Therefore, DPP-4 inhibitors increase the concentrations of GLP-1 and GIP, which, as mentioned above, act in pancreatic β-cells by releasing insulin, and in pancreatic α-cells, inhibiting the secretion of glucagon in a glucose-dependent manner, thus controlling blood glucose with no risk of hypoglycemia. The greatest effect of DPP-4 inhibitors is in the postprandial period, when the levels of glucose are elevated. DPP-4 inhibitors are also known as gliptins. Four gliptins are available: vildagliptin, sitagliptin, saxagliptin, and linagliptin. This antidiabetic class is becoming more important among diabetic patients with CKD, due to their excellent tolerability profile [122–126]. Vildagliptin, sitagliptin, and saxagliptin are excreted by the kidney and require dosage adjustment in patients with creatinine clearance <50 mL/min/1.73 m^2^. For example, vildagliptin, which is used at a dosage of 50 mg twice a day, should be used at the same dose, but as a single daily administration in patients with creatinine clearance <50 mL/min/1.73 m^2^, including patients with Stage 5 CKD [124]. Linagliptin has no renal excretion and therefore does not require adjustment for renal function.

Until recently, the arsenal of noninsulin antidiabetic agents was not safe to be used in diabetic patients with CKD, and insulin therapy was started early, causing psychological distress to patients and families. Nowadays, there are new noninsulin agents, DPP-4 inhibitors in particular, which present a low risk of hypoglycemia and can be used in patients with DM2 with CKD. However, further studies are required to confirm the safety of these new agents in this population. Table 2 summarizes the recommendations for the use of noninsulin antidiabetic agents for noninsulin patients based on international guidelines [70, 103, 104].

**Table 2:** Recommendations for the use of noninsulin antidiabetic agents in CKD

Antidiabetic Agents	Recommendations in CKD
Metformin	With creatinine clearance 30–45 mL/min/1.73 m^2^, halve the dose and suspend the drug when the creatinine clearance is <30 mL/min/1.73 m^2^
Sulfonylureas	Use drugs with a short duration of action and suspend the drugs when the creatinine clearance is <45 mL/min/1.73 m^2^
Glinides	These can be used in patients with CKD, although with care when the creatinine clearance is <30 mL/min/1.73 m^2^
Glitazones (pioglitazone)	Their use is associated with water and salt retention, which limits their use in CKD
Alpha-glucosidase inhibitors (acarbose)	Their use should be avoided in CKD, due to risk of drug accumulation and consequent hepatotoxicity
Sodium-glucose cotransporter type 2 inhibitors	Their use is not indicated with a creatinine clearance <30 mL/min/1.73 m^2^
Peptide-1 receptor agonists similar to glucagon (GLP-1 RA)	Little knowledge in CKD. Gastrointestinal effects are exacerbated in patients with CKD. Use with caution with a creatinine clearance 45–60 mL/min/1.73 m^2^ and avoid its use in patients with a creatinine clearance <45 mL/min/1.73 m^2^
Dipeptidyl peptidase-4 (DPP-4) inhibitors	Low risk of hypoglycemia. These can be used in CKD. With a creatinine clearance <50 mL/min/1.73 m^2^, dosage adjustments should be made for vildagliptin, sitagliptin, and saxagliptin. The dose of linagliptin does not require adjustment in CKD

### Pharmacological treatment of DM in CKD: insulin therapy

**Fig. 3:**
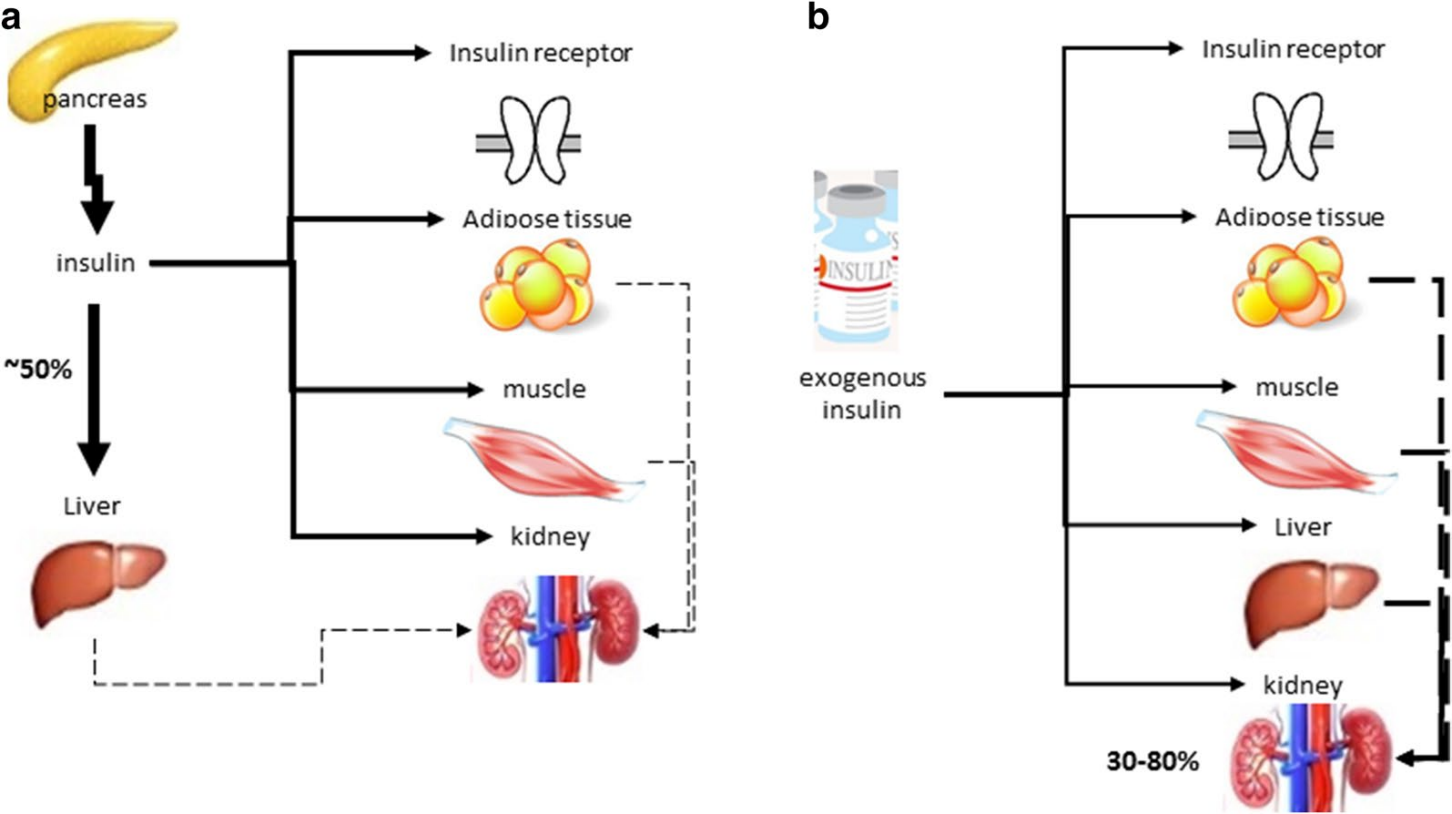
Schematic presentation of the clearance of insulin. **a** endogenous insulin and **b** exogenous insulin. Adapted from Iglesias and Díez [130]

The kidney plays an important role in clearing insulin from the systemic circulation and two distinct pathways have been described; one involves glomerular filtration and subsequent insulin absorption by proximal tubular cells through endocytosis; and the other is related to insulin diffusion through peritubular capillaries and their connection to the contraluminal tubular membrane, especially from the distal half of the nephron. Therefore, insulin is transported by lysosomes and metabolized to amino acids that are released by diffusion in peritubular vessels, and final degradation products are then reabsorbed [127–129]. Endogenous insulin has a mean plasma half-life of only 6 min and it is almost cleared from the circulation within 10–15 min (Fig. 3a). Except for the portion of insulin bound to its receptors on the target cells, the remainder is degraded mainly in the liver, to a lesser extent in kidney and muscle and slightly in most other tissues. In contrast, exogenous insulin does not undergo the first-pass effect in the liver, the kidney plays an important role in the metabolism and clearance of circulating insulin in patients with renal failure (Fig. 3b). As a consequence, with the progression of CKD, insulin clearance decreases, thus requiring a dose reduction in order to avoid hypoglycemia [130, 131].

The pharmacokinetics of commercially available insulin in diabetic patients with reduced glomerular filtration rate has been evaluated for small number of studies. Although the profile of these patients requires less insulin, several authors suggest a reduction of the dose of insulin when GFR is between 10–50 mL/min, around 25 % of total daily dose and 50 % for a GFR <10 mL/min, regardless of the type of insulin used [132].

Insulins are classified according to their action profile (Table 3). Thus, the first exogenous insulins developed to control blood sugar, NPH (Neutral Protamine Hagedorn) and Regular insulin are labeled as having an intermediate- and rapid-acting profile, respectively. One has a peak activity 4–7 h after subcutaneous injection, while the other one is used before meals in order to reduce the peak of hyperglycemia after the ingestion of carbohydrates. However, its onset of action is between 30 min and 1 h and it must be applied around 30–45 min before the meal. The insulin analogs, produced by recombinant DNA technology, are classified as (1) short-acting (lispro, aspart, and glulisine insulin), (2) long-acting (glargine, detemir), or (3) ultra-long-acting (degludec). The association between the short-acting and the long- or ultra-long-acting insulin analogs enables physiological simulation of insulin secretion; this therapeutic association has been termed basal-bolus insulinization.

**Table 3:** Insulin pharmacokinetic profiles

Insulin type	Onset	Peak	Duration of action
Rapid-acting profile			
Regular	30 min	2–4 h	5–7 h
Short-acting profile			
Lispro Aspart Glulisine	5–15 min	60–90 min	3–4 h
intermediate-acting profile			
NPH*	2 h	6–10 h	13–20 h
Long-acting profile			
Glargin	~2 h	Flat	20–24 h
Detemir	~2 h	Less-pronounced peak	6–24 h
Ultra-long-acting profile			
Degludec	20–40 min	Flat	~42 h

Due to its pharmacokinetic profile with a stable half-life and duration of action of about 24 h, glargine insulin can be prescribed once a day. To date, few studies have been published on the use of glargine insulin in patients with renal failure, and its use appears to be safe, with a reduction in HbA1c in a short period of time [133]. In hospitalized patients with a GFR <45 mL/min, the reduction of the dosage calculated according to the body weight was shown to be effective in diminishing the number of hypoglycemic events by 50 %, without compromising metabolic control [134]. Detemir insulin has an onset of drug action of 1 h, and its effect lasts 12–24 h. Thus, it is recommended that this drug be used in two daily doses, with intervals of about 12 h. However, some patients could present different sensitivity along the day, and for this subgroup of patients a single-a-day dose may be enough to maintaining adequate glycemic control in the postprandial period [135, 136]. A recent study [137] demonstrated the need for dose reduction, for both glargine and detemir insulin, in patients with renal function impairment. In this case, the dose of glargine and detemir insulin was 29.7 and 27.3 % lower in individuals with GFR <60 mL/min than in those with GFR >90 mL/min. Degludec insulin, with an ultra-long-action profile, has recently been approved to be commercialized, and only one study in patients with different stages of renal failure and terminal CKD has been published, showing no statistical significant differences in absorption or release profiles when compared to individuals with normal renal function. Thus, degludec insulin does not require dose adjustments due to the loss of kidney function [138].

As shown on Table 3, the insulin analogs lispro, aspart, and glulisine have short durations and very similar pharmacokinetic profiles [139]. Because lispro insulin was the first analog investigated, there are a number of studies in patients with CKD [140–142] showing it has a beneficial effect in reducing glomerular hyperfiltration and renal effects of hyperglycemia triggered by meals; these effects are possibly related to an antagonistic effect on insulin-like growth factor-1 [140]. Furthermore, the use of lispro insulin was associated with improved glycemic control and quality of life in patients on hemodialysis by end-stage diabetic renal disease [141, 142]. The glulisine and aspart insulin also had their safety and efficacy demonstrated in controlling postprandial hyperglycemia in patients with DM2 and severe renal failure [143]. No change in the pharmacokinetic of these drugs was observed [144].

Regardless of insulin being considered the best choice for glycemic control in patients with renal impairment, its prescription must be based on some guidelines, such as: (1) individualization of the therapy; (2) frequent reassessment of prescription or adjustment of doses for the glomerular filtration rate; (3) basal-bolus insulin regimens, prescribing intermediate- or long-acting profile insulin, as basal insulin, to keep the levels of blood glucose stable on post-absorptive period, associated with short-acting profile insulin to promote adequate carbohydrates metabolism and control of postprandial glycaemia; and (4) blood glucose monitoring and frequent adjustment of insulin therapy based on individual response [145]. Few studies have reported specific information on the differences in action profiles, half-life, metabolism, and clearance of different insulin types available that are adjusted for the different stages of CKD; such studies would allow the prescription of more effective therapeutic regimens, minimizing risk of hypoglycemia, which is potentially more harmful in this population. Therefore, the treatment should be individualized based on factors such as the presence of complications, associated diseases, disease management ability, stage and duration of CKD, and previous glycemic control [146–148]. In addition, there should be participation of a multidisciplinary team consisting of nephrologists, endocrinologists, nutritionists, and nurses. This approach has proved to be an effective strategy in achieving individual glycemic optimal values, reducing the rate of progression of kidney disease and other complications associated with DM2, and improving the quality of life of patients with DKD.

## Conclusion

The relationship between DM and DKD is more complicated than the predisposition of a diabetic patient to develop kidney disease and the negative impact on morbidity and mortality of patients with kidney disease and DM. Recently, the kidney has been recognized as being directly involved in the pathogenesis of DM because of its ability to regulate glucose reabsorption as well as to determine insulin half-life and resistance. In addition, it is now clear that glomerular filtration provides a safe and efficacious target for many hypoglycemic drugs. Thus, understanding the renal physiology and pathophysiology of DKD has become essential to all specialties treating diabetic patients. Disseminating this knowledge and detailing the evidence will be important to initiate breakthrough research and to encourage proper treatment of this group of patients.

## Abbreviations

DM: diabetes mellitus
DKD: diabetic kidney disease
DM2: diabetes mellitus type 2
CKD: chronic kidney disease
IR: insulin resistance
GLUTs: glucose transporters of the cell membrane
GLUT4: glucose transporter of the cell membrane 4
HOMA-IR: homeostatic model assessment for insulin resistance
GIP: glucose-dependent insulinotropic peptide
GLP-1: glucagon-like peptide-1
SGLT: sodium/glucose cotransporter
SGLT1: sodium/glucose cotransporter 1
SGLT2: sodium/glucose cotransporter 2
UKPDS: United Kingdom Prospective Diabetes Study
HbA1c: hemoglobin A1c
SM: home glucose monitoring or self-monitoring
HbA: hemoglobin A
NGSP: national glycohemoglobin standardization program
HPLC: high performance liquid chromatography
DCCT: diabetes control and complications trial
EDTA: ethylene diamine tetraacetic acid
HbC: hemoglobin C
HbD: hemoglobin D
HbE: hemoglobin E
HbS: hemoglobin S
GA: glycated albumin
1.5-AG: 1,5-anhydroglucitol
SBD: Brazilian Diabetes Society
ADA: American Diabetes Society
ACCORD: action to control cardiovascular risk in diabetes
ADVANCE: action in diabetes and vascular disease
GFR: glomerular filtration rate
NKF-KDOQI: National Kidney Foundation-Kidney Disease Outcomes Quality Initiative
ACE: angiotensin-converting enzyme
ARBs: angiotensin II receptor blockers
VA-NEPHRON: Veterans Affairs Nephropathy in Diabetes
KDIGO: kidney disease: improving global outcomes
LDL: low-density lipoprotein cholesterol
DR: diabetic retinopathy
DCAN: diabetic cardiovascular autonomic neuropathy
PPAR-ɣ: peroxisome proliferator-activated receptor ɣ
GLP-1 RA: glucagon-like peptide-1 receptor agonist
DPP-4: dipeptidyl peptidase-4
